# miR-145通过下调*OCT4*基因抑制肺腺癌干细胞增殖

**DOI:** 10.3779/j.issn.1009-3419.2011.04.03

**Published:** 2011-04-20

**Authors:** 帅 张, 雅琴 武, 冬杰 冯, 治 张, 峰 蒋, 荣 尹, 林 许

**Affiliations:** 210009 南京，南京医科大学附属江苏省肿瘤医院胸外科 Department of Toracic Surgery, Nanjing Medical University Afliated Cancer Hospital of Jiangsu Province, Baiziting 42, Nanjing 210009, China

**Keywords:** miR-145, 肺癌干细胞, A549细胞株, OCT4, miR-145, Lung cancer stem cells, A549 cell line, *OCT4* gene

## Abstract

**背景与目的:**

miR-145是通过miRNA芯片及qPCR验证筛选出的一种潜在肺癌“保护性”miRNA。本研究旨在探讨miR-145与肺癌干细胞之间的关系及分子机制。

**方法:**

miRNA芯片对肺腺癌患者瘤旁和正常组织进行表达谱分析；生物信息学软件预测miR-145潜在的靶基因；脂质体2000介导转染miR-145模拟物和阻遏物进入A549细胞株；实时定量PCR检测miR-145表达水平；Western blot检测OCT4蛋白水平；双荧光素酶报告基因验证miR-145是否作用于OCT4 mRNA的3’UTR区预测靶位；细胞增殖实验检测miR-145对于A549细胞生长的作用；流式细胞术检测干细胞表型CD133^+^的表达。

**结果:**

在肺腺癌组织中miR-145表达明显低于瘤旁正常组织；miRanda软件预测*OCT4*是miR-145潜在靶基因；与对照组相比，miR-145模拟物组和阻遏物组miR-145表达分别明显上调和下调；miR-145对A549细胞的生长有双向调节作用，过表达miR-145抑制细胞生长；过表达miR-145可明显降低OCT4蛋白水平及干细胞表型CD133百分比，而抑制miR-145表达则明显增加OCT4蛋白水平及CD133百分比。双荧光素酶报告基因检测证明miR-145可作用于OCT4 mRNA的3’UTR区预测靶位。

**结论:**

miR-145可通过下调*OCT4*基因表达抑制A549肺腺癌细胞株中干细胞的增殖，是一种潜在的肺癌“保护性”miRNA。

## Abstract (en)

**Background and objective:**

MiR-145 functions as a protective miRNA identifed in tumor tissues of lung adenocarcinoma patients. The aim of this study is to investigate the relationship between miR-145 and proliferation of lung cancer stem cells and involved molecular mechanisms in human lung adenocarcinaoma A549 cell line.

**Methods:**

MicroRNA microarray technology was conducted to compare miRNA signature between tumor and adjacent normal tissue of lung adenocarcinaoma. The potential target gene of miR-145 was predicted by online bioinformatic sofwares. Pre-miR-145 mimics and anti-miR-145 inhibitor were transfected into A549 cell line by lipofectamine 2000. miR-145 expression in each group was detected by real time PCR. The OCT4 protein level was analyzed by Western blot. The predicted miR-145 binding site in OCT4 3'-untranslated region (UTR) was validated by dual-luciferase reporter gene assay. CCK-8 assay was employed to observe the proliferation activity of A549 cells. The ratio of CD133 positive cells in each group was analyzed by flow cytometry.

**Results:**

miR-145 expression was signifcantly down-regulated in lung adenocarcinoma compared with ajacent normal tissue. OCT4 is a potential target gene of miR-145 predicted by miRanda. Compared with control group, miR-145 was signifcantly up-regulated and down-regulated in the pre-miR-145 mimics and anti-miR-145 inhibitor groups respectively. Overexpression of miR-145 inhibited the proliferation of A549 cells. Both the OCT4 protein level and CD133 positive ratio were remarkably decreased in the pre-miR-145 mimics group, whereas signifcantly increased in the anti-miR-145 inhibitor group. Dual-luciferase reporter gene assay validated the predicted miR-145 binding site of OCT4 3'UTR.

**Conclusion:**

MiR-145 can inhibit the proliferation of lung cancer stem cells in A549 cell line via down-regulating OCT4 expression. MiR-145 is a potential protec-ive miRNA of lung cancer.

已有研究^[1]^证明肿瘤组织中存在一类具有干细胞样潜能的肿瘤发生细胞，它们可能是肿瘤发生、复发和耐药的关键，因而被称为肿瘤干细胞（cancer stem cells, CSCs）。当前，如何遏制CSCs的增殖潜能是肿瘤学领域的研究热点。新近研究^[2]^证明，CD133^+^非小细胞肺癌细胞具有自我更新能力，是肺癌干细胞（lung cancer stem cells, LCSCs）的表面标志之一。

微小RNA（microRNA, miRNA）是一类长度约22个核酸的非编码内源性小RNA，可以与基因mRNA的3’UTR区结合，起到在转录后水平调控基因表达的作用，已有研究^[3]^表明其在调控肿瘤分化、发生、浸润及转移等多个环节发挥重要作用，对未来肿瘤治疗可能产生革命性影响，因而受到广泛重视。我们基于临床肺腺癌组织标本，通过miRNA芯片初筛及qPCR验证发现：肿瘤较正常组织miR-145表达下调明显，提示其是一种潜在的“保护性”miRNA。生物信息学软件预测调控干细胞的关键基因*OCT4*是其可能的靶基因，本研究旨在阐明miR-145在调控LCSCs增殖中的作用及可能的分子机制。

## 1 材料与方法

### 1.1 试剂及细胞株

miRNA表达谱分析采用美国LC Science公司μParaflo^TM^ microRNA微流体微阵列芯片。miRNA提取试剂盒mirVana RNA Isolation Kit，逆转录试剂盒Taqman miRNA Reverse Transcripation Kit，荧光实时定量PCR试剂盒Taqman Universal PCR Master Mix No AmpErase UNG和内参RNU6B MGB探针标记的引物购于美国ABI公司。miR-145模拟物Pre-miR-145 mimics、阻遏物Anti-miR-145 inhibitor、miR-145模拟物阴性对照Pre-miR-145 mimics negative control和阻遏物阴性对照Anti-miR-145 inhibitor control购于美国ABI公司。转染试剂Lipofectamine 2000购于Invitrogen公司。肺腺癌A549及用于转染质粒载体的Hela细胞株购于中国科学院上海细胞生物学研究所细胞库，RPMI1640培养基购于Hyclone公司，胎牛血清购于Gibco公司。OCT4、β-actin一抗购于Abcam公司，HRP标记的二抗购于北京中杉金桥生物科技有限公司，CD133一抗购于Cell Signaling Technology公司，FITC标记的二抗购于美国KPL公司。双荧光素酶报告基因检测试剂盒购于美国Promega公司。CCK-8试剂盒购于南京凯基生物有限公司。

### 1.2 miRNA表达谱分析

随机选择临床上10例肺腺癌患者的手术标本，男女比例为1:1。Trizol法提取样品总RNA，通过YM-100（Millipore）微离心过滤柱得到片段小于300 nt的小RNA，参照Sanger 13.0 miRNA数据库，进行μParaflo^TM^ miRNA微流体微阵列芯片检测。杂交检测使用Cy3和Cy5特异性荧光标记、激光扫描仪（GenePix 4000B, Molecular Device）采集杂交图象、Array-Pro（Media Cybernetics）软件对杂交图像进行数字化转换。数据处理和分析首先是扣除背景，计算重复点平均值和标准偏差，然后通过LOWESS（Locally-Weighted Regression）过滤进行标准化。在10例标本中，共同上调或下调*P* < 0.01的差异表达miRNA被初筛用以进一步研究。

### 1.3 miRNA的real time PCR相对定量检测

检测对象为20例随机选择的的肺腺癌患者肿瘤及瘤旁正常组织标本及在转染后48 h收获的细胞样品，RNA的提取、逆转录和PCR反应均严格按照ABI公司提供的Taqman miRNA assay的方法，以RNU6B作为内参进行相对定量，每组设置3个复孔，在ABI 7500实时定量荧光PCR仪机器检测。

### 1.4 生物信息学软件靶基因预测

应用在线生物信息学miRNA靶基因预测软件miRand（网址http://www.miRBase.org/）对miR-145的靶基因进行预测。

### 1.5 细胞培养和转染

肺腺癌A549细胞株培养在RPMI1640培养液+10%胎牛血清中，温度37 ℃，CO_2_浓度5%。将A549细胞分为5组，即miR-145模拟物转染组，阻遏物转染组，模拟物阴性对照转染组，阻遏物阴性对照转染组和只加转染试剂的空白对照组。转染在六孔细胞培养板内、待细胞结合度约50%-70%时进行，每孔板加入25 pmol的模拟物和10 μL的转染试剂，使模拟物的终浓度为10 pmol/mL，转染后5 h换液。

### 1.6 Western blot蛋白水平检测

在转染后48 h收获细胞，以β-actin为内参，步骤按照常规方法，见文献^[4]^。采用Image J软件对条带的灰度值进行分析。

### 1.7 双荧光素酶报告基因检测

克隆扩增*OCT4*基因的3’ UTR区的全长，引物序列：forward primer 5’ GGTGCCTGCCCTCTAGGAATG3’，reverse primer 5’ TAAGTGTGTC TATCTACTGTGT3’，重组到psiCHECK-2，生物信息学预测mir-145与*OCT4*基因的靶结合位点，并针对该点进行定点突变，表达海肾荧光素酶的pRL-TK载体用来作为内参照调整细胞数量和转染效率的差异，miR-145模拟物以及阴性对照和miR-145阻遏物及其阴性对照分别和火萤荧光素酶报告载体共转染进入Hela细胞，双荧光素酶活性检测严格按照Promega提供的方法^[5]^。

### 1.8 细胞增殖活力检测

采用CCK-8方法检测转染细胞的增殖活力，分别在转染1 d-6 d内检测。细胞接种在96孔板内，每孔内加入5, 000个细胞，100 µL培养液和10 µL检测试剂。于添加CCK-8试剂2 h后上酶标仪在450 nm处检测各孔OD值，每组重复4次。相对增殖活力=处理组OD值/空白对照组OD值。

### 1.9 流式细胞术检测CD133^+^表型

在转染后72 h收获细胞，采用间接标记方法。流式细胞仪为FACS Calibu（美国BD公司），分析软件为Flowjo 7.6版本。

### 1.10 统计学方法

统计软件采用SPSS 13.0版本。数据结果以均数±标准差的方式显示，多组间均数比较采用单因素方差分析（ANOVA），*P* < 0.05认为有统计学差异，数据图表采用Graphicpad Prism 5.0软件制作。

## 2 结果

### 2.1 miRNA芯片表达谱分析及real time PCR验证

经过PCR验证，共有9个miRNA在肿瘤组织中表达明显下调（包括miR-126、miR-223、miR-451、miR-145、miR-26b、miR574-5p、miR26b、let-7b、let-7c，*P* < 0.05），除已有研究的let-7家族和miR-126等miRNA外，miR-145的表现引人关注，miR-145在几乎所有患者肿瘤组织中均明显下调（*P* < 0.01），且70%患者差异表达3倍以上（图 1）。

**1:**
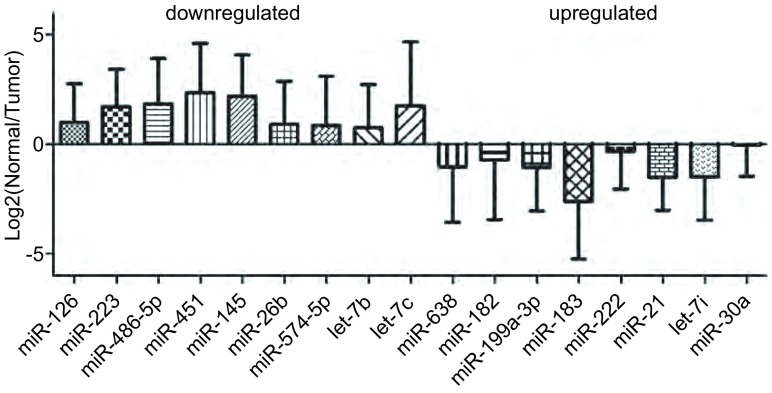
Real time PCR对芯片分析的验证结果。图中各组miRNA表达数值为Log2（正常组织/肿瘤组织），其中miR-145在几乎所有患者肿瘤组织中均显著下调（*P* < 0.01）。 Real time PCR validation of miRNAs screened by microarray

### 2.2 生物信息学软件预测

通过对miRNA数据库的查询，找到miR-145的成熟序列和其种子序列，运用在线预测软件miRanda对其靶基因进行分析，其中*OCT4*为其可能的靶基因（图 2）。

**2:**
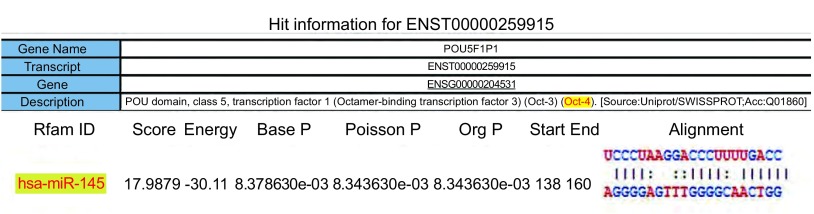
miRanda在线生物信息学软件预测miR-145靶基因。（POU5F1P1即*OCT4*基因） Potential targeting gene of miR-145 predicted by miRanda online bioinformatics software

### 2.3 Real time PCR检测各组miR-145的表达水平

miR-145模拟物转染组的miR-145表达明显上调，是空白对照组的13.78±0.38倍（*P* < 0.001），阻遏物转染组miR-145的表达水平明显下调，是空白对照组的0. 1 1 ± 0. 0 1倍（*P* < 0.001），阴性对照组与空白对照组之间的miR-145表达无明显统计学差异（图 3）。表明miR-145模拟物明显促进了A549细胞中miR-145的表达，而miR-145阻遏物则明显抑制miR-145的表达。

**3:**
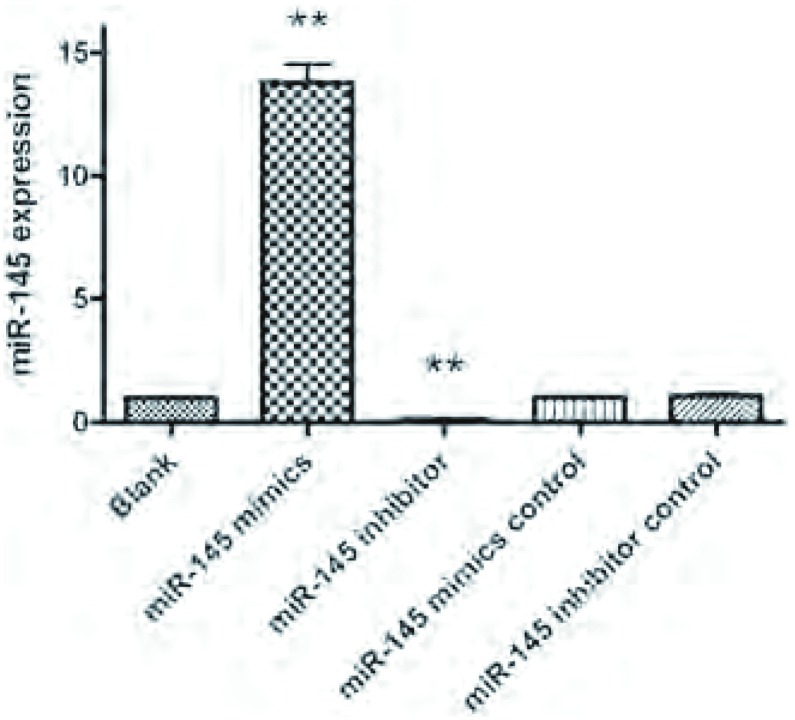
Real time PCR检测各转染组miR-145的表达情况。图中以空白对照组（Blank）为基准，表达水平为1.00。^**^P < 0.001 *vs*空白对照组。 The expression of miR-145 in each group determined by real time PCR. The blank group was considered as baseline and its value was 1.00. ^**^P < 0.001 *vs* blank group.

### 2.4 OCT4蛋白水平

miR-145模拟物转染组和miR-145阻遏物转染组中OCT4蛋白表达较其他三组有明显差异，OCT4蛋白在模拟物转染组中表达明显下调，而在阻遏物转染组中表达明显上调（图 4）。说明miR-145可能抑制OCT4蛋白的表达。

**4:**
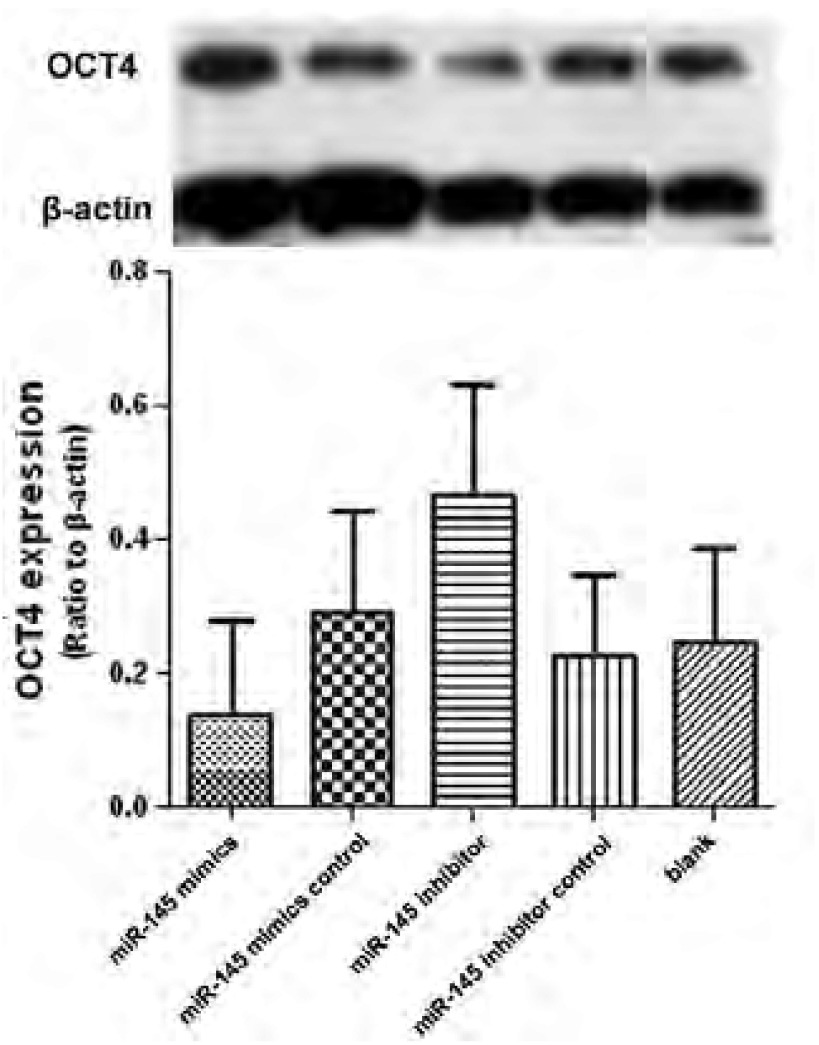
Western blot检测各转染组的蛋白水平 OCT4 protein level determined by western blot

### 2.5 双荧光素酶报告基因检测

对miR-145和OCT4的野生型结合位点进行定点突变。miR-145模拟物和野生型OCT4共转染组的Hela细胞荧光素酶活性下降约50%（*P* < 0.001），而突变型转染组中荧光素酶活性无明显变化，同时在miR-145阻遏物和阴性对照组中也未观察到有荧光素酶活性的改变（图 5），统计分析显示差异无统计学意义。该结果表明miR-145可以直接调控*OCT4*基因。

**5:**
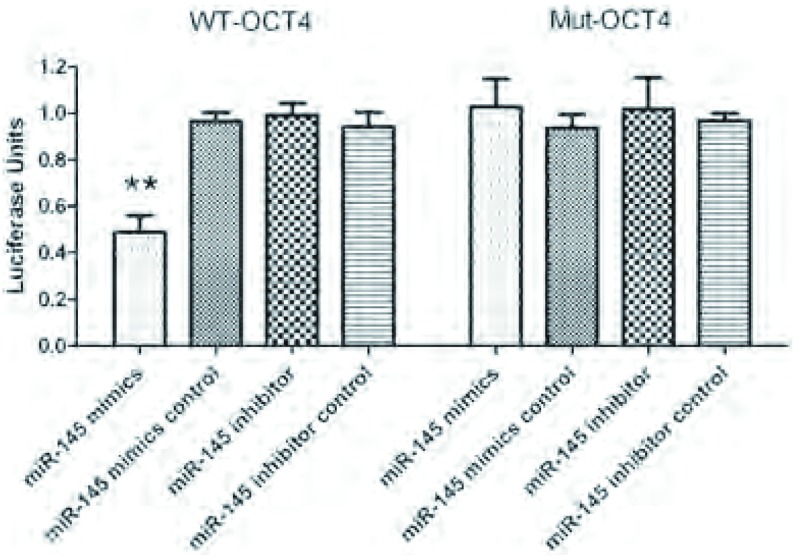
双荧光素酶报告基因检测结果。^**^P < 0.001。 Dual-luciferase reporter gene assay. ^**^P < 0.001.

### 2.6 细胞在转染后1 d-6 d内的增殖活力

如图 5所示，细胞在转染miR-145模拟物后增殖活力变强，在转染miR-145阻遏物后增殖缓慢（*P* < 0.01，图 6），其他三组增殖活力差异无明显统计学意义（*P* < 0.01）。同时细胞增殖活性改变在转染后3 d-4 d时表现最明显。该结果表明miR-145可以抑制A549细胞的增殖。

**6:**
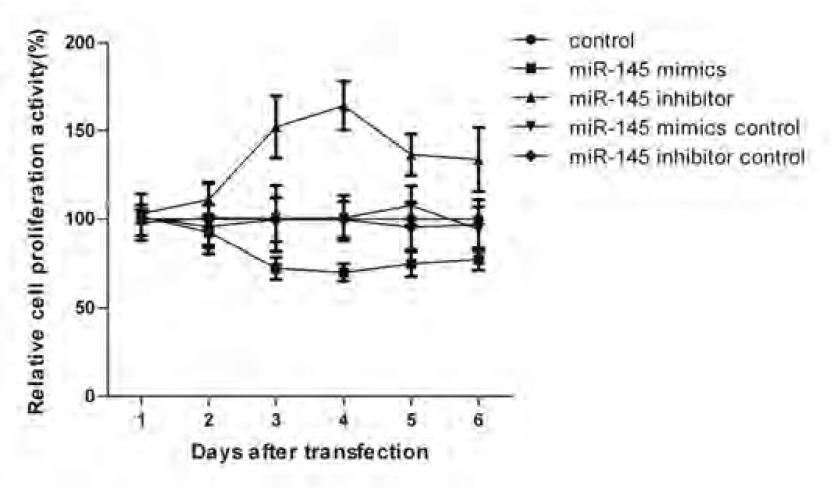
CCK-8试验检测细胞增殖活力 Cell proliferation assay by CCK-8 kit

### 2.7 CD133^+^表型分析

如图 7所示，CD133^+^细胞的比率在miR-145模拟物组为4.53%±0.37%，与模拟物对照组6.50% ±0.92%相比明显降低（*P* < 0.05）；而miR-145阻遏物组为9.86%±1.50%，与阻遏物对照组6.62%±0.74%相比明显升高（*P* < 0.01）；miR-145模拟物组与miR-145阻遏物组亦明显降低（*P* < 0.01）。表明miR-145可明显抑制LCSCs的增殖。

**7:**
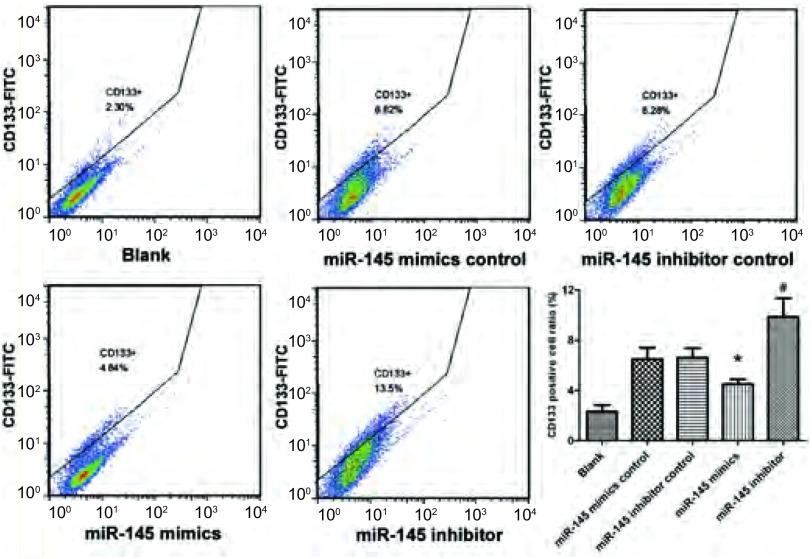
流式细胞仪检测CD133^+^表型细胞的比率。^*^P < 0.05 *vs* miR-145模拟物阴性对照组；^#^*P* < 0.01 *vs* miR-145阻遏物阴性对照组。 CD133^+^ cell ratio analyzed by flow cytometer. ^*^P < 0.05 *vs* miR-145 mimics control group; ^#^*P* < 0.01 *vs* miR-145 inhibitor control group.

## 3 讨论

miRNA是现今肿瘤学的研究热点之一，能与多种靶基因结合且具有可预测性的特点尤其吸引人们的注意。越来越多的文献^[7]^表明miRNA可以作为一项工具来调控与肿瘤发生发展有关的信号通道和一些关键基因或因子，并将与肿瘤有关的这一类miRNA称为oncomir。目前，与肺癌相关的miRNA研究已取得了一些进展，但目前明确对肺癌发生发展具有调控作用的miRNA仍需继续发掘，因此采用高通量技术筛选与肺癌相关的差异miRNA，对其进行功能学研究仍十分必要。

参照Sanger 13.0 miRNA数据库，我们随机选取肺腺癌患者组织标本进行了miRNA表达谱分析以及real time PCR验证，筛选出17种肿瘤与正常组织相比具有明显表达差异的miRNA，例如let-7家族、miR-126和miR-145等明显下调，这与Yanaihara等^[8]^的研究结果相似。除已有研究的let-7家族和miR-126等miRNA外，miR-145的表现引人关注，miR-145在几乎所有患者肿瘤组织中均明显下调。为深入研究肺癌中miR-145的作用，我们通过文献检索发现：虽已有报道^[9]^在乳腺癌中miR-145表达显著下调，并可抑制乳腺癌细胞增殖，但miR-145在肺癌中的生物功能学研究尚未见报道。因此，我们利用TargetScan、miRanda等在线分析软件，来预测miR-145可能的下游靶基因，发现其中包含维持干细胞自我更新特性的关键基因之一*OCT4*^[10]^。在胚胎干细胞（embryonic stem cells, ES）研究^[11]^中，miR-145可调控OCT4等控制细胞自我更新潜能的关键转录因子基因，从而抑制ES的分化；而OCT4等ES特异性标志在各种实体肿瘤中亦呈现高表达，在肺癌中OCT4的表达越高，预后越差^[12]^。因此OCT4等核转录因子可能是抑制CSCs无限分化潜能的关键基因。

为了阐明miR-145和*OCT4*基因之间的关系，我们对体外生长的肺腺癌A549细胞株进行miRNA模拟物和阻遏物的转染分别促进和抑制miR-145的表达，运用western blot和双荧光素酶报告基因的技术从蛋白和基因两个水平来验证两者之间靶向调节作用，最终证明miR-145可以直接调控*OCT4*基因。我们的研究还表明miRNA与靶基因的结合具有相对特异性，miR-145对于野生型的*OCT4*基因具有显著的调控的作用，但对于突变型的*OCT4*基因却未表现出靶向调控作用。

我们通过细胞增殖实验对转染miR-145的A549细胞进行功能学研究发现，过表达的miR-145可以抑制A549细胞的生长，因此我们进一步在机制方面进行了探索。目前，肿瘤干细胞学说的提出为人们对肿瘤基础研究和临床干预提供了一条新的思路，鉴定、分离和靶向调控干细胞成为研究热点。鉴于*OCT4*基因在人类干细胞分化中扮演的重要角色，Chen等^[13]^研究也证明*OCT4*基因的表达是维持CD133^+^表型特征的原因之一。因此，其表达的变化也会影响CSCs的分化。我们针对LCSCs特异性表面标志CD133进行了流式细胞术检测，以此来评价LCSCs的增殖状况。研究结果表明miR-145可以明显地调节A549细胞株中CD133^+^表型细胞的比率，而这种作用的实现很可能是通过miR-145对*OCT4*基因的调控来实现的。

综上，通过调控干细胞自我更新关键基因*OCT4*从而抑制LCSCs增殖可能是miR-145抑制肺腺癌生长的重要机制。由于CSCs是肿瘤发生发展的恶性源头，如果能调控甚至逆转肿瘤干细胞的生长分化必将对肿瘤的治疗带来深远影响。因此，本研究为肺癌的生物学防治提供了一个新的潜在靶标。
